# Identification, classification and evolution of Owl Monkeys (*Aotus*, Illiger 1811)

**DOI:** 10.1186/1471-2148-10-248

**Published:** 2010-08-12

**Authors:** Albert N Menezes, Cibele R Bonvicino, Hector N Seuánez

**Affiliations:** 1 Departamento de Genética, Universidade Federal do Rio de Janeiro, Cidade Universitária - 21949-570 Rio de Janeiro, RJ, Brazil; 2 Programa de Genética - Instituto Nacional de Câncer, Rua André Cavalcanti, 37 - 4° andar, 20231-050 Rio de Janeiro, RJ, Brazil; 3 Laboratório de Biologia e Parasitologia de Mamíferos Reservatórios Silvestres, Instituto Oswaldo Cruz, Rio de Janeiro, RJ, Brazil

## Abstract

**Background:**

Owl monkeys, belonging to the genus *Aotus*, have been extensively used as animal models in biomedical research but few reports have focused on the taxonomy and phylogeography of this genus. Moreover, the morphological similarity of several *Aotus *species has led to frequent misidentifications, mainly at the boundaries of their distribution. In this study, sequence data from five mitochondrial regions and the nuclear, Y-linked, *SRY *gene were used for species identification and phylogenetic reconstructions using well characterized specimens of *Aotus nancymaae*, *A. vociferans*, *A. lemurinus*, *A. griseimembra*, *A. trivirgatus*, *A. nigriceps*, *A. azarae boliviensis *and *A. infulatus*.

**Results:**

The complete *MT-CO1*, *MT-TS1*, *MT-TD, MT-CO2*, *MT-CYB *regions were sequenced in 18 *Aotus *specimens. ML and Bayesian topologies of concatenated data and separate regions allowed for the proposition of a tentative *Aotus *phylogeny, indicating that *Aotus *diverged some 4.62 Million years before present (MYBP). Similar analyses with included GenBank specimens were useful for assessing species identification of deposited data.

**Conclusions:**

Alternative phylogenetic reconstructions, when compared with karyotypic and biogeographic data, led to the proposition of evolutionary scenarios questioning the conventional diversification of this genus in monophyletic groups with grey and red necks. Moreover, genetic distance estimates and haplotypic differences were useful for species validations.

## Background

**Figure 1:**
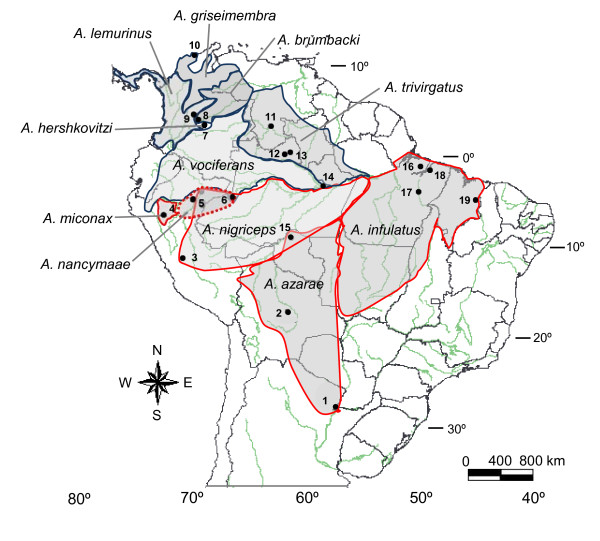
**Geographic distribution of *Aotus *species, from Hershkovitz **[10]**with modifications**. The range of *A. nancymaae*, partially overlapping with *A. nigriceps *and *A. vociferans *is shown by a red/stippled line. Map shows type localities (place where holotype or type specimen was found) and sites of collection. Type localties: 1 = Argentina, right bank of Rio Paraguay (*A. a. azarae*); 2 = Bolivia, Prov. de Sara (*A. a. boliviensis*); 3 = Perú, Chanchamayo; 4 = Perú, San Nicolas; 5 = Perú, right bank of Rio Samiria; 6 = Brazil, Tabatinga; 7 = Colombia, East side of Cordillera; 8 = Colombia, Villavicencio; 9 = Colombia, Santa Fé de Bogotá; 10 = Colombia, Hacienda Cincinnati; 11 = Venezuela, Duida Range; 18 = Brazil, Belém do Pará. Sites of collection: 6 = Colombia, Letícia; 12 = Brazil, Santa Isabel do Rio Negro; 13 = Brazil, Barcelos, 14 = Brazil, Manaus; 15 = Brazil, Usina Hidroeletrica de Samuel; 16 = Brazil, Ilha de Marajó, 17 = Brazil, Usina Hidroeletrica de Tucuruí; 19 = Brazil, São Miguel.

The small-sized neotropical primates with unique nocturnal habits, known as "owl monkeys" or "night monkeys" are grouped in the genus *Aotus*. This genus is widespread across several biomes of South America, and in Panama at the northwestern part of its distribution (Figure 1). Several *Aotus *species have been extensively used as animal models for vaccine research, vision physiology and susceptibility to viral infections but few reports have focused on the taxonomy and phylogeography of this genus. Moreover, several surveys on the distribution of neotropical primates have not included owl monkeys because their nocturnal habit makes them elusive to field workers [1,2].

Early taxonomic studies of *Aotus *were mainly based on pelage coloration [3] and karyotyping [4-8], leading to the revision of the taxonomic status of several species [9] and the description of new ones [10]. In Hershkovitz's taxonomic revision, two novel species were described, comprising a total of nine species divided in two groups with different pelage coloration (red neck and grey neck). *Aotus *taxonomy and evolution has also been analyzed based on morphologic, karyologic and carbonic anhydrase II electrophoretic data [11,12] while phylogenetic reconstructions were later inferred based on partial cytochrome oxidase COII DNA data [13,14]. A study of grey neck species [15], mainly based on karyotypic data, recognized at least seven species of this group, *A. brumbacki*, *A. griseimembra*, *A. lemurinus*, *A. trivirgatus*, *A. vociferans*, *A. zonalis *and a presumably novel one named *A. jorgehernandezi*.

The morphological similarity of *Aotus *species has led to frequent misidentifications, mainly at the borderlines of their distributions where more than one species might be captured at collecting sites. Care should be taken when selecting specimens for taxonomic and phylogenetic studies although the karyotype of most *Aotus *species is well known and might be useful for their identification. In fact, precise identification is crucial for biomedical studies using *Aotus *as animal models. Several studies suggested that grey neck species were susceptible to malaria contrary to the presumably resistant red neck species [10,11] although the red neck species *A. azarae boliviensis *and *A. infulatus *were found to be susceptible to *Plasmodium falciparum *[16,17] while different *Plasmodium *species or strains showed varying degrees of pathogenicity among *Aotus *species. Similarly, different *Aotus *species displayed manifold phenotypes to HIV-1 restriction by tetherin [18], an integral membrane protein that prevents budding of nascent retroviral particles in infected cells [19]. Tetherin alleles from *A. nancymaae *and *A. vociferans *potently restricted HIV-1 replication while those from *A. griseimembra *failed to do so, showing that closely related species might express different restriction phenotypes [20]. These findings are illustrative of the need of precise species identification for validating biomedical research.

Here we report a study of *Aotus *with five mitochondrial DNA sequences and one Y-linked gene and used these markers for species identification and phylogenetic reconstructions. This allowed us to reassess several GenBank specimens, analyze the species status of *Aotus *taxa and infer putative evolutionary scenarios based on biogeographic and karyotypic data.

## Methods

### Samples

We analyzed 18 *Aotus *specimens belonging to eight *Aotus *taxa and two *Saimiri sciureus *(Table 1). Figure 1 shows the geographic distribution of *Aotus *species and sites of collection. Skull and skins of *Aotus trivirgatus *(TR1 and TR2; field numbers CRB1479 and CRB2597, respectively) were deposited in the mammal collections of Museu Nacional (MN), Universidade Federal do Rio de Janeiro, Rio de Janeiro, Brazil (catalog numbers MN69297 and MN53667, respectively).

**Table 1:** List of specimens herein sequenced, with identification (ID), field number, species, sex, place of collection and GenBank accession number

ID	Species	Field Number	Sex	Place of collection	GenBank *MT-CO1 *to *MT-CO2*	GenBank *MT-CYB*	GenBank *SRY*
AB1	*A.a. boliviensis*	17954*	♂	Samuel, Rondônia, BR	[GenBank:HQ005472]	[GenBank:HQ005492]	[GenBank:HQ018923]
AB2	*A.a. boliviensis*	17956*	♀	Samuel, Rondônia, BR	[GenBank: HQ005473]	[GenBank:HQ005493]	
IN1	*A. infulatus*	AI1*	?	São Miguel, Maranhão, BR	[GenBank: HQ005474]	[GenBank:HQ005494]	
IN2	*A. infulatus*	AI3*	♂	São Miguel, Maranhão, BR	[GenBank: HQ005475]	[GenBank:HQ005495]	[GenBank: HQ018924]
IN3	*A. infulatus*	AI4*	♂	Ilha do Marajó, Pará, BR	[GenBank: HQ005476]	[GenBank:HQ005496]	
NI1	*A. nigriceps*	10174	♀	São Paulo Zoo, São Paulo, BR	[GenBank: HQ005478]	[GenBank:HQ005498]	
NI2	*A. nigriceps*	CPRJ2203	♂	CPRJ, Rio de Janeiro, RJ	[GenBank:HQ005477]	[GenBank:HQ005497]	[GenBank:HQ018925]
LE1	*A. lemurinus*	ALL01	♂	UFPA, Pará, BR	[GenBank:HQ005486]	[GenBank:HQ005506]	[GenBank:HQ018927]
GR1	*A. griseimembra*	M43	♂	San Marcos, Colombia	[GenBank:HQ005485]	[GenBank:DQ098872]**	
GR2	*A. griseimembra*	D13	♂	San Marcos, Colombia	[GenBank:HQ005484]	[GenBank:DQ098870]**	[GenBank:AF338374]**
TR1	*A. trivirgatus*	CRB1479*	♀	Rio Aracá, Amazonas, BR^1^	[GenBank:HQ005480]	[GenBank:DQ098873]**	
TR2	*A. trivirgatus*	CRB2597*	♂	Rio Padauiri, Amazonas, BR^2^	[GenBank:HQ005481]	[GenBank:DQ098874]**	
TR3	*A. trivirgatus*	18440*	♂	Manaus, Amazonas, BR	[GenBank:HQ005479]	[GenBank:HQ005499]	[GenBank:HQ018926]
VO1	*A. vociferans*	AV3032*	?	Letícia, Colombia	[GenBank:HQ005483]	[GenBank:HQ005503]	
VO2	*A. vociferans*	AV3051*	♂	Letícia, Colombia	[GenBank:HQ005482]	[GenBank:HQ005502]	[GenBank:HQ018928]
NA1	*A. nancymaae*	AN3033*	?	Letícia, Colombia	[GenBank:HQ005488]	[GenBank:HQ005508]	
NA2	*A. nancymaae*	AN3056*	?	Letícia, Colombia	[GenBank:HQ005487]	[GenBank: HQ005507]	
NA3	*A. nancymaae*	AN3058*	?	Letícia, Colombia	[GenBank:HQ005489]	[GenBank:HQ005509]	
SA1	*Saimiri sciureus*	JAO1975*	?	Brazil	[GenBank:HQ005491]	[GenBank:HQ005511]	
SA2	*Saimiri sciureus*	CRB1780*	?	Brazil	[GenBank:HQ005490]	[GenBank:HQ005510]	

? = unknown; * = wild caught; ** = previously reported sequences; 1 = 0°32'N, 63°31'W; 2 = 0°18'N, 64°01'W; BR = Brazil.

### DNA isolation, amplification and sequencing

DNA was extracted from blood or liver tissue preserved in ethanol following standard procedures [21]. Cytochrome *b *DNA was amplified with primers L14724 [22] and Citb2 [23], and amplified products were labeled with primers Citb AloAotR [24] and Citb AloAotF [24], Citb Alo [23] and Citb Aot (5'-CATGAGGCCAAATATCATTCTGAGG-3'). Cytochrome Oxidase Units I and II were amplified with two primer pairs: CO1F/CO1R (5'-ATGCTTACTCAGCCATTTTA-3' and 5'-TTTGAGGAGAAAGCTTCTC-3' respectively) and CO1F3/CO2R2 (5'-TTGCTATCCCTACTGGGGTAAA-3' and 5'-GGTCTTTAACTTAAAAGGTTAATGCTARRTA-3' repectively). Amplified products were sequenced with primers CO1R2 (5-AATGGCTCCTAGAATTGAAGAAA-3), CO2F1 5' CTCCTCCTTATCACACATTT 3') and CO2F2 (5' TAACYCACACCAGCACCATA 3'). *SRY *DNA was amplified with primers SRY- [25] and SW2 [26]; internal primers SRY 2i- and SRY 2i+ were used for sequencing [25]. Cytochrome *b *DNA of *A. griseimembra *and *A. trivirgatus *has been previously reported [20].

All fragments were amplified under the following conditions: 94° (3 min); 35 cycles of 94° (30 sec), 55° (45 sec), 72° (90 sec); and a final extension of 72° (3 min). Amplified products were purified using Illustra GFX PCR DNA and Gel Band Purification Kit (GE Healthcare). Sequencing was carried out with ABI Prism™ 377 and ABI 3730 DNA Analyzers, manually aligned with Chromas Lite version 2.01 [27] and Bioedit [28] and deposited in GenBank.

Nomenclature of mitochondrial DNA sequences were *MT-CO1 *for cytochrome *C *oxidase subunit I, *MT-TS1 *for tRNA serine 1, *MT-TD *for tRNA aspartic acid, *MT-CO2 *for cytochrome *C *oxidase subunit II, *MT-CYB *for cytochrome *b*, and *SRY *for the Y chromosome "sex determining region Y" gene following HGNC rules (latest accession on January 2010 [29,30]).

### Analyses of molecular data

Separate analyses were carried out with the following DNA datasets: (1) Dat-CON, with concatenated *MT-CO1, MT-CO2 *and *MT-CYB *sequences; (2) Dat-CO1, with *MT-CO1 *sequences; (3) Dat-CO2, with *MT-CO2 *sequences; (4) Dat-CYB, with *MT-CYB *sequences. (5) Dat-SRY, with *SRY *sequences. A partition homogeneity test [31] implemented in PAUP* 4.0b10 [32] was performed using 1,000 replications and 100 random addition replicates to compare phylogenetic signals between different data partitions in Dat-CON.

A complete mitochondrial DNA sequence of *Cebus albifrons *([GenBank:AJ309866]) and *MT-CO1, MT-TS1, MT-TD, MT-CO2, MT-CYB *data from two *Saimiri sciureus *sequenced by us (SA1 and SA2) were used as outgroups as was the *SRY *DNA sequence of *Cebus albifrons *([GenBank:AF338385]).

The best model of evolution for each dataset was estimated with the Akaike information criterion test [33] with modifications (AIC2) [34] and the Bayesian Information Criterion (BIC) [35] with Modelgenerator 0.85 [36]. Estimates of sequence divergence were calculated and constructed with pairwise deletion using PAML 4.4 [37] except for Dat-SRY due the low number of variable sites. Maximum likelihood (ML) trees were obtained with PAUP* 4.0b10 [32] by heuristic searches with the tree-bisection-reconnection algorithm (TBR) and 100 random addition sequences. Additionally, 1,000 bootstrap replicates indices were obtained with GARLI version 0.96 [38] (available in http://garli.nescent.org). Bayesian posterior probabilities were computed using the Metropolis-coupled Markov chain Monte Carlo method (MCMCMC) with MrBayes 3.1.1 [39], by running four chains with 20,000,000 generations. Trees were sampled every 100 generations and the first 10% were discarded as the "burn-in" phase before computing a consensus tree with PAUP*. Bayesian posterior probabilities (PP) were obtained from the 50% majority rule consensus of the remaining trees. Bayesian analyses for Dat-CON were performed in a partitioned framework, allowing specific parameter estimation for each locus. Comparisons of alternative topologies (Additional file 1) were carried out with BASEML of PAML 4.4 package [37].

The time of divergence between species was estimated with Dat-CON using a Bayesian Markov chain Monte Carlo (MCMC) algorithm with BEAST 1.5.3 [40], with unlinked substitution and unlinked relaxed clock [41] models for each gene. We used 21.81 ± 1.24 Million years before present (MYBP) as the time of divergence of *Aotus *from *Cebus*/*Saimiri*, and 19.05 ± 1.5 MYBP as the time of divergence of *Cebus *from *Saimiri *[42], with a normal prior distribution, a randomly generated starting tree and chain length of 20,000,000 generations with parameter samples every 1,000 steps. Monophyletic taxon sets were assumed for congruence with topologies produced by ML and Bayesian phylogenetic reconstructions. For acceptable mixing and convergence to the stationary distribution, the first 10% were discarded as burn-in using Tracer [43] and TreeAnnotator 1.5.4 of BEAST 1.5.3 [40] package.

Additionally, GenBank sequences from several *Aotus *specimens (Table 2) were compared with analogous datasets (Dat-CO1, Dat-CO2 and Dat-CYB). ML and Bayesian phylogenetic reconstructions were carried out using the same parameters as above, although branch lengths were not estimated because several GenBank specimens contained only partial sequence data. Only *Cebus albifrons *was used as outgroup in the analysis of *MT-CO2 *sequences from GenBank.

**Table 2:** GenBank specimens, DNA sequenced region, size in base pairs (bp), position of the first nucleotide respective to our sequence data (1^st ^bp = first bp)

GenBank	DNA sequence	bp	**1**^**st **^**bp**	Identification in GenBank and publications	Identification according to position in topologies
[GenBank:AY250707]	All MT*	3528	1	*A. trivirgatus*	*A. griseimembra*
[GenBank:AJ309866]	All MT*	3528	1	*Cebus albifrons*	-
[GenBank:EF658652]	*MT-CO1*	629	58	*A. azarae*	Not confirmed
[GenBank:EF658653]	*MT-CO1*	629	58	*A. azarae*	Not confirmed
[GenBank:EF658654]	*MT-CO1*	629	58	*A. azarae*	Not confirmed
[GenBank:EF658655]	*MT-CO1*	629	58	*A. azarae*	Not confirmed
[GenBank:EF658656]	*MT-CO1*	629	58	*A. azarae*	Not confirmed
[GenBank:EU179516]	*MT-CO1*	651	53	*A. azarae*	Not confirmed
[GenBank:EU179517]	*MT-CO1*	649	53	*A. azarae*	Not confirmed
[GenBank:AY972694]	*MT-CO1*	614	58	*A. nancymaae*^(1)^	confirmed
[GenBank:AF352254]	*MT-CO2*	696	1	*A. nancymaae*	confirmed
[GenBank:AF352255]	*MT-CO2*	696	1	*A. nancymaae*	confirmed
[GenBank:AF352256]	*MT-CO2*	696	1	*A. nigriceps*	confirmed
[GenBank:AF352257]	*MT-CO2*	696	1	*A. nigriceps*	confirmed
[GenBank:AF352258]	*MT-CO2*	696	1	*A. nigriceps*	confirmed
[GenBank:AF352259]	*MT-CO2*	696	1	*A. vociferans*	confirmed
[GenBank:AF352260]	*MT-CO2*	696	1	*A. vociferans*	confirmed
[GenBank:U36770]	*MT-CO2*	646	21	***A. nancymaae***^(2)^	confirmed
[GenBank:U36843]	*MT-CO2*	621	41	***A. l. griseimembra***^(2)^	confirmed
[GenBank:U36844]	*MT-CO2*	602	41	***A. l. griseimembra***^(2)^	confirmed
[GenBank:U36845]	*MT-CO2*	585	58	***A. l. griseimembra***^(2)^	confirmed
[GenBank:U36846]	*MT-CO2*	568	58	***A. a. boliviensis***^(2)^	Not confirmed
[GenBank:DQ321659]	*MT-CO2*	549	28	*A. nancymaae*^(3)^	confirmed
[GenBank:DQ321660]	*MT-CO2*	549	28	*A. nancymaae*^(3)^	confirmed
[GenBank:DQ321661]	*MT-CO2*	549	28	*A. l. griseimembra*^(3)^	?
[GenBank:DQ321664]	*MT-CO2*	549	28	*A. nigriceps*^(3)^	*A. trivirgatus*
[GenBank:DQ321665]	*MT-CO2*	549	28	*A. vociferans*^(3)^	confirmed
[GenBank:DQ321666]	*MT-CO2*	549	28	*A. vociferans*^(3)^	confirmed
[GenBank:DQ321669]	*MT-CO2*	549	28	*A. brumbacki*^(3)^	?
[GenBank:DQ321670]	*MT-CO2*	549	28	*A. a. azarae*^(3)^	*A. nigriceps*
[GenBank:AJ489745]	*MT-CYB*	1140	1	*A. nancymaae*^(4)^	*A. griseimembra*
[GenBank:AJ489746]	*MT-CYB*	1140	1	*A. nancymaae*^(4)^	*A. griseimembra*
[GenBank:AF338385]	*SRY*	832	1	*Cebus albifrons*	-
[GenBank:AF181085]	Numt**	696	1	*A. a. azarae*^(5)^	Not confirmed

(1) Lorenz et al. [61]; (2) Ashley and Vaughn [13]; (3) Plautz et al. [14]; (4) Lavergne et al. [62]; (5) Ascunce et al. [63]; * = mtDNA including *MT-CO1 *to *MT-CO2 *and MT-CYB; ** = Nuclear mitochondrial DNA insertion. Karyotypic data were reported for species shown in bold.

Aminoacid sequences were deduced, for *MT-CO1*, *MT-CO2*, *MT-CYB *and the coding region of *SRY *(nt 177 - 803) using MEGA 4.02 [44]. The best models of evolution for each deduced protein were obtained using the same parameters as above. ML topologies with 1,000 bootstrap replicates were constructed with PHYML 3.0 [45] and Bayesian phylogenetic reconstructions were carried out as previously described.

### Karyotypic analyses

Cell suspensions of *A. nigriceps *NI2 were prepared with short-term cultures of 46 hours in Dulbecco's minimal essential medium, enriched with fetal serum (20%), phytohemagglutinin (2%), and colchicine (10^-6 ^M) during the last two hours. Short term, bone marrow cultures of TR1 female specimen of *A. trivirgatus *from Barcelos were prepared in the field and incubated for 2-hours in RPMI 1640 medium, fetal calf serum (20%), colchicine (10^-6 ^M) and ethidium bromide (5 μg/mL).

## Results

### Gene markers

The complete *MT-CO1*, *MT-TS1*, *MT-TD, MT-CO2*, *MT-CYB *genes were sequenced in 18 *Aotus *specimens belonging to eight taxa (Table 1) and *Saimiri sciureus *SA1 and SA2 except for the initial 9 bp region of the *MT-CO1 *5'-region of SA2. A region containing five *SRY *regions (832 pb) was also sequenced: (1) the untranslated region upstream of the start codon (nt 1-176), (2) the codon region upstream of the HMG box (nt 177-350), (3) the HMG box domain (nt 351-582), (4) the downstream coding region (nt 583-800), (5) the untranslated downstream region (nt 801-829) from at least one specimen of the following species: *A. azarae boliviensis*, *A. infulatus*, *A. nigriceps*, *A. trivirgatus, A. vociferans, A. lemurinus *and *A. griseimembra *(Table 1). The size of each Dataset, number of specimens, haplotypes, variable sites, parsimony informative sites, number of protein variable sites and number of deduced proteins are listed in Table 3. Genetic distance estimates are shown in Table 4.

**Table 3:** Comparisons of different datasets, dataset size (in base pairs = bp), number of *Aotus *specimens, number of taxa, number of haplotypes, variable sites (in DNA), parsimony informative sites per dataset, number of variable sites in deduced proteins, and number of different proteins in coding regions per individual gene dataset

Dataset	Size (bp)	Number of specimens	Number of taxa	Number of haplotypes	Variable sites (DNA)	Parsimony Informative Sites	Variable sites (protein)	Number of proteins
Dat-CON	3,396	18	8	18	505	434	-	-
Dat-CO1	1,560	18	8	17	210	182	8	8
Dat-CO2	696	18	8	15	85	66	2	3
Dat-CYB	1,140	18	8	18	210	181	36	17
Dat-SRY	834	7	7	7	12	5	4	4

**Table 4:** Intraspecific and interspecific genetic distance estimated with the HKY + G model in each dataset showing lower and higher estimates or single estimates.

Species	Species	Dat-CO1	Dat-CO2	Dat-CYB	Dat-CON
*A. a. boliviensis*	*A. a. boliviensis*	**.004**	**0**	**.008**	**.005**
	*A. infulatus*	.007-.010	.004-.006	.013-.021	.008-.013
	*A. nigriceps*	.021-.023	.017	.055-.061	.031
	*A. trivirgatus*	.061-.068	052-.054	.079-.089	.066-.071
	*A. vociferans*	.064-.068	.055	.084-.091	.069-.072
	*A. nancymaae*	.062-.069	.053-.059	.081-.095	.069-.074
	*A. griseimembra*	.062-.065	.042-.045	.078-.087	.064-.067
	*A. lemurinus*	.066	.046	.085-.093	.068-.070
*A. infulatus*	*A. infulatus*	**0-.004**	**.001-.009**	**.002-.016**	**.001-.009**
	*A. nigriceps*	.021-.024	.017-.020	.060-.064	.031-.034
	*A. trivirgatus*	.061-.066	.050-.059	.080-.090	.066-.070
	*A. vociferans*	.065-.067	.054-.060	.088-.093	.071-.072
	*A. nancymaae*	.062-.070	.052-.063	.089-.101	.071-.076
	*A. griseimembra*	.064-.069	.045-.049	.079-.093	.065-.072
	*A. lemurinus*	.066-.069	.048-.050	.086-.098	.069-.074
*A. nigriceps*	*A. nigriceps*	**.002**	**0**	**.005**	**.003**
	*A. trivirgatus*	.060-.066	.048-.051	.088-.099	.067-.071
	*A. vociferans*	.066-.068	.047	.094-.098	.070-.072
	*A. nancymaae*	.056-.063	.050-.056	.096-.104	.069-.072
	*A. griseimembra*	.058-.060	.044-.046	.097-.103	.067-.070
	*A. lemurinus*	.065	.045	.096-.097	.070-.071
*A. trivirgatus*	*A. trivirgatus*	**.001-.006**	**0-.004**	**.001-.015**	**.001-.008**
	*A. vociferans*	.064-.069	.058-.060	.068-.073	.065-.068
	*A. nancymaae*	.062-.068	.054-.059	.067-.077	.065-.068
	*A. griseimembra*	.058-.062	.052-.055	.066-.076	.060-.064
	*A. lemurinus*	.062-.064	.059	.070-.074	.063-.067
*A. vociferans*	*A. vociferans*	**.002**	**.001**	**.003**	**.002**
	*A. nancymaae*	.042-.047	.041-.048	.064-.072	.051-.053
	*A. griseimembra*	.044-.046	.036-.040	.060-.065	.049-.050
	*A. lemurinus*	.047-.049	.043-.044	.063-.065	.052-.053
*A. nancymaae*	*A. nancymaae*	**.001-.007**	**.002-.006**	**.010-.015**	**.004-.009**
	*A. griseimembra*	.044-.051	.040-.046	.066-.068	.052-.055
	*A. lemurinus*	.045-.050	.047-.051	.068-.072	.055
*A. griseimembra*	*A. griseimembra*	**.003**	**.002**	**.004**	**.003**
	*A. lemurinus*	.038-.042	.026-.028	.023-.024	.031-.033

Intraspecific values are shown in bold

Comparisons with *Cebus albifrons SRY *([GenBank:AF338385]) showed a minimum of 45 nucleotide substitutions and one deletion in all *Aotus *corresponding to nucleotides 672 to 674 of *C. albifrons*.

### Phylogenetic analyses of DNA sequences

The partition homogeneity test did not show significant differences between loci (p = 0.116; *p *≥ 0.05) supporting the congruence and subsequent combination of the three mitochondrial genes for ML analysis. Also, the Hasegawa-Kishino-Yano model [46] with Gamma shape parameter (HKY + G) was indicated by AIC2 and the BIC tests for phylogenetic reconstructions using all mitochondrial sequences datasets. For *SRY *analysis, both tests indicated the Kimura's 2-parameter model (K80) [47].

**Figure 2:**
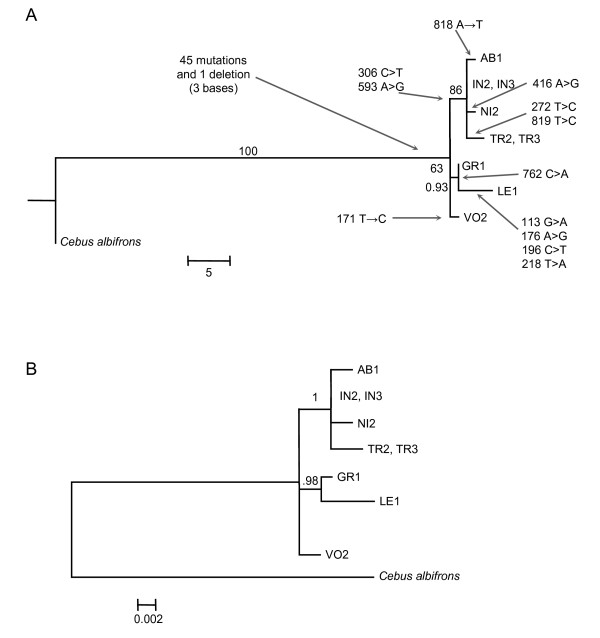
**Topologies derived from Dat-SRY analyses**. A) ML topology obtained with heuristic search, 100 random addition sequence and K80 model. Numbers near nodes correspond to bootstrapping frequencies ≥ 60 estimated with 1,000 replicates. Arrows indicate *SRY *mutations (base pair position and nucleotide substitutions). B) 50% majority rule consensus topology of 18,000 sampled trees. Numbers at nodes indicate Bayesian proportions.

The ML topology resulting from analysis of *SRY *data (Figure 2) showed three collapsed lineages: one leading to *A. vociferans*, a second one leading to *A. griseimembra *and *A. lemurinus*, and a third one leading to *A. trivirgatus A. nigriceps A. azarae boliviensis*, and *A. infulatus*.

**Figure 3:**
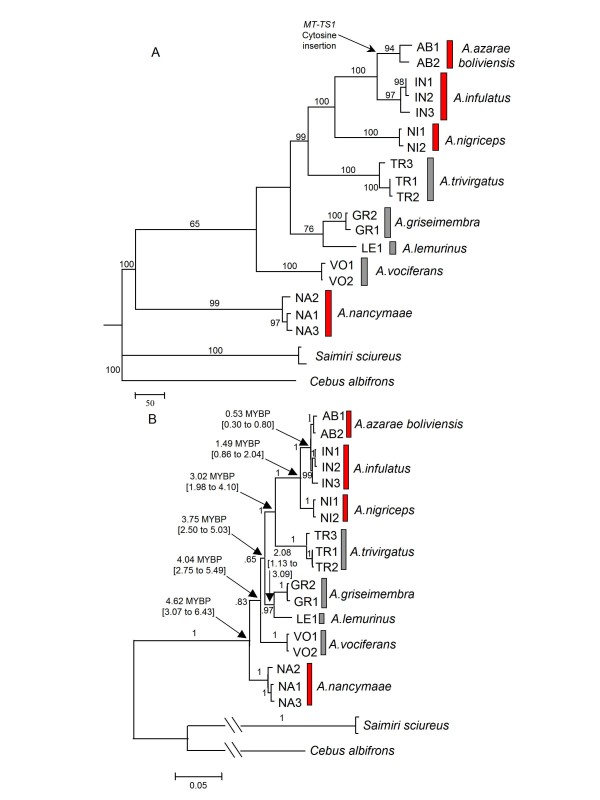
**Concatenated genes topologies**. Red bars and grey bars indicate red neck and grey neck species, respectively. A) ML topology, with heuristic search, HKY + G model; 100 random addition sequence. Numbers correspond to bootstrapping frequencies ≥ 60 estimated with 1,000 replicates; Arrow indicates Cytosine insertion in *MT-TS1*. B) 50% majority rule consensus topology of 18,000 sampled trees. Numbers at nodes indicate Bayesian proportions. Arrows indicate divergence times in MYBP (Height and 95% height posterior density intervals). Lineages leading to *Cebus albifrons *and *Saimiri sciureus *were reduced for better resolution of *Aotus *lineages.

ML and Bayesian reconstructions using Dat-CON showed two sister lineages (Figure 3A-B), one leading to the most basal offshoot represented by *A. nancymaae *and another to a clade grouping the seven other *Aotus *taxa. This clade split in two sister lineages, one leading to *A. vociferans *and the other one further splitting in (*A. griseimembra, A. lemurinus*) and to a more derived clade (*A. trivirgatus *(*A. nigriceps *(*A. infulatus, A. azarae*))).

Two ML topologies resulting from analysis of Dat-CO1 and the consensus topology (Additional file 2A) showed a similar arrangement to the one resulting from Dat-CON analysis, except that NA2 did not group with the other *A. nancymaae *specimens. Bayesian reconstruction (Additional file 2B) was identical with the Dat-CON topology.

The ML topology produced by analysis of Dat-CO2 (Additional file 3A) showed few differences with the one produced by Dat-CON, with a paraphyletic arrangement of *A. infulatus *while, in the Bayesian reconstruction (Additional file 3B), specimen IN3 grouped with *A. azarae boliviensis *(AB1 and AB2) rather than with other *A. infulatus *(IN1 and IN2). Moreover, the branches leading to *A. vociferans*, *A. lemurinus *and *A. griseimembra *collapsed in the Bayesian topology.

The ML topology derived from Dat-CYB (Additional file 4A) showed two clades, one grouping (*A. vociferans *(*A. trivirgatus *(*A. nigriceps *(*A. azarae boliviensis, A. infulatus*)))), and another grouping (*A. nancymaae *(*A. griseimembra, A. lemurinus*)) while, in the Bayesian reconstruction (Additional file 4B) the branches leading to *A. vociferans *and *A. nancymaae *collapsed, as was also the case of the branch leading to *A. griseimembra *and *A. lemurinus*.

Comparisons between different topologies using Dat-CON, carried out with BASEML are shown in Additional file 1. These trees were constructed taking in consideration the differences between the following topologies: (1) Dat-CON ML (Figure 3A); (2) Dat-CO2 Bayesian (Additional file 3B); (3) Dat-CYB ML (Additional file 4A); (4) Dat-CYB Bayesian (Additional file 4B); (5) Dat-CON ML (Figure 3A) with collapsed low supported lineages (*A. nancymaae*, *A. vociferans*, *A. lemurinus *and *A. griseimembra*); (6) Dat-CO2 ML (Additional file 3A) with collapsed low supported lineages (*A. nancymaae*, *A. vociferans*, *A. lemurinus*/*A. griseimembra *and *A. trivirgatus*). Topology 1 showed the highest RELL bootstrap proportion (pRELL = 0.79) although topologies 3 and 4 could not be rejected by the Shimodaira and Hasegawa test [48] (p-values = 0.62 and 0.48 respectively) despite showing lower pRELL (0.19 and 0.01 respectively). Conversely, topologies 2, 5 and 6 where discarded (p-values = 0.02, 0.01 and 0.03 respectively).

### Phylogenetic analyses of deduced protein sequences

The AIC2 and BIC test indicated the MtMam model [49] with invariable sites parameter (MtMam+I) as the best model phylogenetic reconstructions based on MT-CO1 and MT-CO2 protein data, while MtMam with Gamma shape parameter (MtMam+G) was indicated for MT-CYB, and the Jones-Taylor-Thornton (JTT) [50] model for SRY. Analyses of aminoacid sequences (Additional file 5) showed different arrangements and less supported nodes than their respective ML nucleotide topologies. Comparisons of protein and nucleotide ML topologies showed the following differences: (1) *A. infulatus *(IN2), *A. azarae boliviensis *(AB1), *A. nigriceps *(NI2) and *A. trivirgatus *(TR3) shared the same SRY protein sequence; (2) *A. azarae boliviensis *(AB1, AB2) and *A. infulatus *(IN1, IN2, IN3) shared the same MT-CO1 protein sequence, as was the case of *A. griseimembra *(GR1, GR2) with *A. trivirgatus *(TR3), and of *A. vociferans *(VO1) with *A. nancymaae *(NA2). On the other side, another *A. vociferans *(VO2) appeared as the most basal lineage; (3) *A. trivirgatus *(TR1, TR2, TR3) shared the same MT-CO2 protein sequence, *A. azarae boliviensis *(AB1, AB2), *A infulatus *(IN1, IN2, IN3), *A nigriceps *(NI1, NI2), *A lemurinus *(LE1), *A griseimembra *(GR2), *A vociferans *(VO1, VO2) and A. *nancymaae *(NA1, NA2, NA3) shared another protein sequence while one A. *griseimembra *(GR1) showed a third protein. Furthermore, the topology did not group *Saimiri *with *Cebus*; (4) the MT-CYB protein arrangement showed a clade (*A. trivirgatus *(*A. vociferans *(*A. lemurinus *(*A. griseimembra*)))) with *A. nancymaae *specimens as three basal lineages.

### Analysis of GenBank data

**Figure 4:**
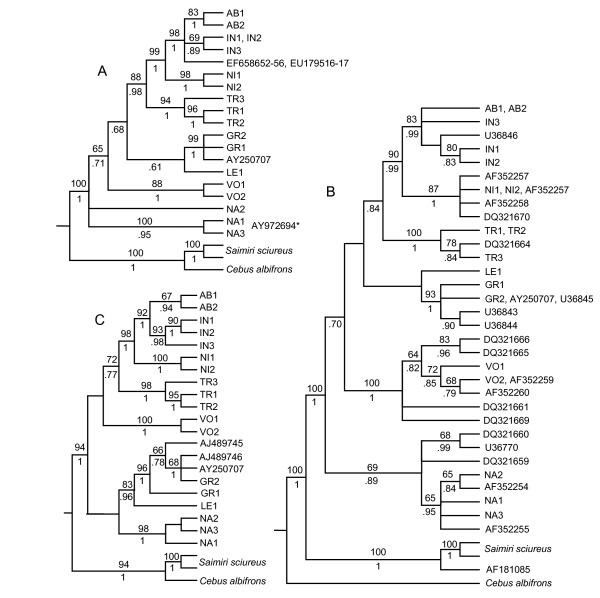
**Topologies resulting from analyses of Dat-CO1, Dat-CO2 and Dat-CYB with included GenBank sequences**. ML topologies, with heuristic search, HKY + G model; 100 random addition sequence. Numbers correspond to bootstrapping frequencies ≥ 60 estimated with 1,000 replicates. Numbers below nodes indicate Bayesian proportions of 18,000 sampled trees. A) Dat-CO1 with included GenBank data; GenBank sample AY972694 shares an *MT-CO1 *region with both NA1 and NA3; B) Dat-CO2 with included GenBank data; C) Dat-CYB with included GenBank data.

ML topologies resulting from the inclusion of GeneBank specimes with Dat-CO1 (Figure 4A), Dat-CO2 (Figure 4B) and Dat-CYB (Figure 4C) show the position of GenBank specimens. A comparison between GenBank identification and identification based on topologic positions is listed in Table 2.

### Karyotypic analyses

Karyotypic analysis of the male specimen NI2 showed a diploid number (2n) of 51 chromosomes corresponding to the diploid and arrangement of *A. nigriceps *Karyotype VII previously described by Ma et al. [8]. Analysis of the *Aotus trivirgatus *female showed 2n = 50; the chromosome complement containing 12 pairs of biarmed chromosomes varying in size from large to small, and 13 pairs of acrocentric chromosomes varying in size from medium to small.

## Discussion

### Molecular markers and species identification

Our findings showed different *MT-CO1*, *MT-CO2*, *MT-CYB *and *SRY *haplotypes between *Aotus *species although some *SRY *haplotypes differed by only one nucleotide (see Figure 2A), as was the case of *A. infulatus *(IN2) when compared to *A. azarae boliviensis *(AB1) and *A. nigriceps *(NI2).

Identification based on *MT-CO2 *was shown to be unreliable for closely related species in view of (i) small gene size, (ii) relative paucity of parsimony informative sites, and (iii) low genetic distance between species.

Species identification of several GenBank specimens was confirmed while the identification of other specimens was reassessed (see Table 2). The identification of 7 *A. azarae *specimens ([GenBank:EF658652] - [GenBank:EF658656, GenBank:EU179516, GenBank:EU179517]) and one *A. azarae boliviensis *([GenBank:U36846]) could not be confirmed because only partial sequence data were available, lacking phylogenetic resolution. Specimen [GenBank:DQ321669], reported as *A. brumbacki*, could not be confirmed because only partial sequence data were available and a reference specimen was not available for comparison. Finally, specimen [GenBank:AF181085], reported as *A. a. azarae*, with a presumably complete *MT-CO2 *sequence, grouped with *Saimiri sciureus*. This sequence presented several sites with missing or ambiguous data and lacked a stop codon, suggesting a nuclear mitochondrial DNA insertion (Numt).

### Phylogenetic considerations

Analyses of the different datasets showed that Dat-CO1 contained the highest number of PI sites (182), closely followed by Dat-CYB (181), while Dat-CO2, showed the lowest number (66). This might be related to the smaller size of *MT-CO2 *(696 bp) respective to *MT-CO1 *(1,557 bp) and *MT-CYB *(1,140 bp) although a similar number of PI sites was found in these two last genes regardless of their difference in size. These findings, and the fact that several *MT-CO2 *intraspecific distance estimates were higher than interspecific estimates, and lack of resolution between *A. azarae *and *A. infulatus*, indicated that *MT-CO2 *was the least reliable marker for analyzing *Aotus *phylogeny. Thus, phylogenetic reconstructions based on *MT-CO2 *[13,14] must be carefully reconsidered.

Phylogenetic reconstructions resulting from Dat-CON provided the most coherent topologies (ML and Bayesian), with strong support for most branches while analysis of *MT-CO1 *resulted in very similar phylogenetic reconstructions, only differing by the position of NA2 in the ML topology (Additional file 2A). In the MT-CO1 protein topology (Additional file 5B), however, the shared sequence between *A. griseimembra *and *A trivirgatus *pointed to the close relation of these two grey neck species although *A. lemurinus *failed to group with *A. griseimembra*. On the other hand, the MT-CO1 protein shared by the red neck species *A. nancymmae *and the grey neck species *A. vociferans *indicated a close relation between them. Analyses of *MT-CO2 *showed paraphyletic arrangements in both ML and Bayesian topologies (Additional file 3) while the MT-CO2 protein topology (Additional file 5C) could only distinguish *A. trivirgatus *species apart from the others. The *MT-CYB *ML topology (Additional file 4) differed from all other DNA topologies by showing a weakly supported grouping of *A. griseimembra*/*A. lemurinus *with *A. nancymaae *(two grey neck species with one red neck species). The MT-CYB protein topology (Additional file 5D) showed one cluster of red neck species, a second cluster grouping all grey neck species, and placed the red neck *A. nancymaae *specimens as basal offshoots.

The monophyly of *Aotus *was corroborated by a single three base deletion in *Aotus *respective to *Cebus SRY *(Figure 2). *SRY *from *A. vociferans *(VO2), *A. griseimembra *(GR2) and *A. lemurinus *(LE1) shared two synapormophies (C and A at positions 306 and 593, respectively) with *Cebus albifrons*. The grouping of the red neck species *A. nigriceps, A. infulatus, A. azarae boliviensis *with the grey neck species *A. trivirgatus *in the *SRY *topology and the common SRY protein sequence shared by these species (Additional file 5A) coincided in showing their close relation.

### Evolutionary, karyological and taxonomic considerations

**Figure 5:**
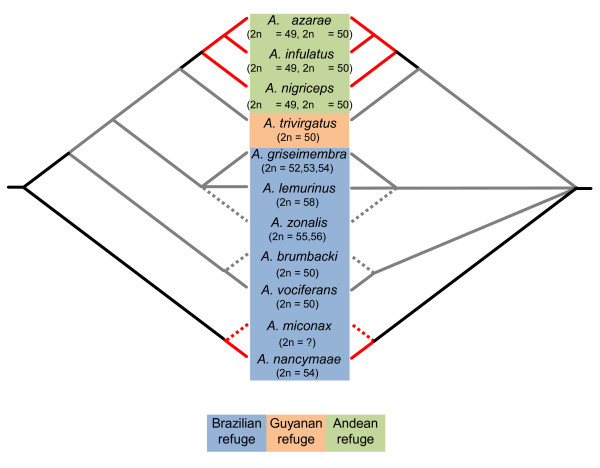
**Proposed scenarios of *Aotus *evolution**. Left: Tentative *Aotus *phylogeny as indicated by Dat-CON analyses based on 8 *Aotus *species herein analyzed. The presumptive positions of *A. miconax*, *A. brumbacki *and *A. zonalis *are based on biogeographic and karyotypic data [11,15]. Diploid chromosome number (2n) is indicated. This arrangement requires at least two separate events accounting for red neck pelage. Right: Alternative polytomy resulting from collapsed branches. This arrangement also requires at least two separate events accounting for red neck pelage.

Our phylogenetic reconstructions question the classical grouping of red neck and grey neck species proposed by Hershkovitz [10]. All phylogenetic reconstructions based on mtDNA showed that the lineage leading to the grey neck species *A. trivirgatus *was a sister lineage of the one leading to the red neck clade (*A. nigriceps *(*A. azarae*, *A. infulatus*)). If the proposed topologies were accepted as a valid representation of the evolutionary divergence of *Aotus *the red neck pelage trait must had appeared independently in *A. nancymaae *and in the red neck clade (*A. nigriceps *(*A. azarae*, *A. infulatus*)) and this would also be acceptable in the event of an alternative polytomy (Figure 5) which might be proposed in view of the low value of bootstrap and posterior probability indices at some nodes (Figure 3, Additional files 2 and 3). Moreover, phylogenetic reconstructions based on SRY and MT-CO1 protein sequence data showed close relations between red neck and grey neck species; this latter topology as well as the MT-CYB topology (Additional file 5D) corroborated that the red neck trait was not monophyletic. On the other hand, the monophyly of the grey neck group was only apparent in the MT-CYB topology, albeit with low support.

Lack of *SRY *data from *A. nancymaae *does not allow us to infer the position of this species using this nuclear marker. Analyses of carbonic anhydrase II biochemical patterns, karyology and morphology suggested that *Aotus *split in two separate clades, one comprising the red neck species and another with the grey neck species [11]. Cytonuclear discordances, as the ones resulting from differences between nuclear and mtDNA phylogenic reconstructions might well result from retention of ancestral polymorphisms during a short time of evolutionary divergence or reticulate evolution represented by historic episodes of hybridisation.

Biogeographic data indicate that red neck species are distributed south of the Solimões-Amazonas River and grey neck species north of this river except for the red neck *A. nancymaae *and the grey *A. vociferans*, the only *Aotus *species occurring north and south this river, and in sympatry in some localities [10] (Figure 1). Contrary to the proposition of Hershkovitz [10], that *A. nancymaae *emerged south of the Solimões-Amazonas, originating all other red-neck species and migrating to the north of this river, our phylogenetic reconstructions indicate the alternative possibility that *A. nancymaae *might have emerged north of the Solimões-Amazonas, originating the grey-neck group, in agreement with the proposed geographic origin of the ancestral *Aotus *population [11].

Altogether, the genus *Aotus *comprises a suite of karyotypically rearranged species consequently to extensive shuffling of their chromosome complement respective to other neotropical primates and man. This has been demonstrated by reciprocal chromosome painting between humans and *A. nancymaae *as well as with *A. nancymaae *chromosomes painted with wooly monkey whole chromosome probes [51]. This study showed that *A. nancymaae *karyotype that only three human syntenic groups were conserved, coexisting with 17 derived human homologous associations, while a minimum of 14 fissions and 13 fusions were required to derive the *A. nancymaae *karyotype from that of the ancestral karyotype of neotropical primates.

The monophyly of the red neck species *A. azarae, A. infulatus *and *A. nigriceps *was in agreement with karyologic data showing that they shared the same X_1_X_1_X_2_X_2_/X_1_X_2_Y sex chromosome system, contrary to other species with an XX/XY sex chromosome system (Figure 5). In all analyses based on DNA sequence data, the grey neck *A. trivirgatus *was the closest species to this red neck group, suggesting that *A. azarae*/*A. infulatus*/*A. nigriceps *might have originated from a more recent common ancestor with *A. trivirgatus *in eastern Amazonia, rather than in western Amazonia from an older common ancestor with *A. nancymaae *(a species with an XX/XY sex chromosome system). Determining the sex chromosome system of *A. trivirgatus *might be elucidating for a better understanding of this scenario.

*Aotus trivirgatus *showed 2n = 50 one female (TR1) from Barcelos (Amazonas State, Brazil), at the southwestern limit of its distribution (Figure 1). Recently, a new *Aotus *species, *A. jorgehernandezi*, has been described based on karyotypic data of a female specimen of unknown provenance [15,52] with the same diploid number as the *Aotus trivirgatus *karyotyped by us. Other specimens collected near Manaus, with 2n = 52 in one female, 2n = 51 in one male, and 2n = 51 or 52 in another male, were previously attributed to *A. trivirgatus *[53]. However, as the distribution of *Aotus *species were not clearly delimited, their identification is questionable; in fact Manaus (Figure 1) is located at the confluence of the distribution of *A. vociferans*, *A. nigriceps *and *A. trivirgatus*.

*Aotus *specimens captured in Samuel Hydroeletric dam reservoir, the same locality of our *A. azarae boliviensis *specimes, showed a 2n♂ = 49; 2n♀ = 50 karyotype [54]. The karyotypic similarity between *A. infulatus *and *A. azarae *suggested a close proximity and recent common ancestry, a finding coincident with their low interspecific distance estimates and their unresolved arrangement when analyzing Dat-CO2, and by the recent time of their evolutionary divergence (0.53 MYBP; Figure 2B).

*Aotus azarae boliviensis *separated from *A. infulatus *when analysing Dat-CON, Dat-CO1 and Dat-CYB (Figures 2, Additional files 2 and 4); the insertion of one cytosine in position 59 of *MT-TS1 *in all *A. azarae boliviensis *being exclusive of this species. These differences as well as the presence of different *SRY *haplotypes justify the status of *A. infulatus *as a valid species rather than a junior synonym of *A. azarae *[55]. This taxonomic arrangement implies that the eastern distribution of *A. azarae *is limited by the rivers Tapajós-Juruema. We agree with Silva Jr & Fernandez [56] and Silva Jr et al. [57] in considering the *A. infulatus *distribution from southeast Amapá, north of the Amazonas River and the Islands of the mouth of the Amazonas to south of this river, from the Rio Tapajós, in the west, to the left bank to Rio Parnaíba in the east (Figure 1).

*Nictipithecus felinus *von Spix, 1823 was considered a junior synonym of *A. infulatus *by Elliot [58] and a junior synonym of *A. trivirgatus *by Groves [55]. *Nictipithecus felinus *was first described with an ochraceus neck (similar to *A. infulatus *and unlike the grey neck *A. trivirgatus*) and illustrated by von Spix [59] in his original description, while the illustration of *Nictipithecus vociferans *showed a grey neck. The *N. felinus *holotype was collected in "les environs de la capitale de Pará", or nearby Belém, the capital of Pará state (Brazil). These findings indicated that *Nictipithecus felinus *was a junior synonym of *A. infulatus*, a reason why we restrict the type locality of *A. infulatus *to Belém, Pará state, Brazil.

Our findings indicated that *A. lemurinus*, *A. griseimembra *and *A. vociferans *are valid species. This is because genetic distance estimates between *A. lemurinus *and *A. griseimembra *were higher than many other interspecific estimates and even higher when comparing *A. vociferans *with *A. lemurinus *and *A. griseimembra *(Table 4), and because *A. lemurinus *and *A. griseimembra *differed by more *SRY *replacements than between any two sister lineages in our ML topology (see Figure 2). These findings argue against the proposition that *A. lemurinus *and *A. griseimembra *are junior synonyms of *A. vociferans *[11] and in agreement with a recent karyologic study [15] indicating that these species are valid taxa.

Our findings indicated that the genus *Aotus *diverged some 4.62 MYBP (with 95% HPD intervals of 3.07 - 6.43 MYBP), and probably before the previous estimate of 3.3 MYPB [13]. Ma [60] suggested that geographic isolation in geographic niches led to karyotypic diversity in *Aotus*. According to Plautz et al. [14], the 100m rise of sea level over the past 5 million years probably allocated *Aotus *species in three refuge groups, one comprising *A. vociferans*, *A. lemurinus*, *A. griseimembra *in the Andean foothills, *A. trivirgatus *in the northwestern Guyanan shield, and *A. nigriceps*, *A. azarae, A. infulatus *and *A. nancymaae *in the Brazilian shield refuge. Our findings suggested that *A. nancymaae *should be included in the Andean foothill refuge rather than in the Brazilian shield refuge (Figure 5) and that *A. nigriceps*, *A. azarae *and *A. infulatus *must have diverged after the rise of sea level while grey neck species and *A. nancymaae *could have diverged before this event.

## Conclusions

Our findings provided a tentative phylogenetic reconstruction of *Aotus *and considered an alternative polytomy based on the low support of some critical nodes. Regardless of these alternatives, however, comparisons with biogeographic and karyotypic data led to the proposition of an evolutionary scenario that questioned the classical division of *Aotus *in grey and red neck groups. Comparisons of GenBank sequences with data from well characterized specimens were useful for assessing the identification of deposited specimens. Our findings further indicated that *A. infulatus*, *A. lemurinus*, *A. griseimembra *and *A. vociferans *are valid species based on genetic distances and haplotypic differences.

## Authors' contributions

ANM designed this study, carried out molecular experiments and analyses, and drafted the manuscript. CRB participated in designing this study and contributed to the analysis of data and drafting the manuscript. HNS revised the original and final version of the manuscript. All authors read and approved the final manuscript.

## Supplementary Material

Additional file 1 **Six topologies compared with BASEML**. Click here for file

Additional file 2 **Topologies derived from Dat-CO1 analyses**. A) ML topology, with heuristic search, HKY + G model; 100 random addition sequence. Numbers correspond to bootstrapping frequencies ≥ 60 estimated with 1,000 replicates. The lineage leading to NA2 collapses while NA1 and NA3 are grouped. B) 50% majority rule consensus topology of 18,000 sampled trees. Numbers at nodes indicate Bayesian proportions. NA1, NA2 and NA3 are grouped. Click here for file

Additional file 3 **Topologies derived from Dat-CO2 analyses**. A) ML topology, with heuristic search, HKY + G model; 100 random addition sequence. Numbers correspond to bootstrapping frequencies ≥ 60 estimated with 1,000 replicates. IN3 is paraphyletic respective to IN1 and IN2. B) 50% majority rule consensus topology of 18,000 sampled trees. Numbers at nodes indicate Bayesian proportions. IN3 is paraphyletic respective to IN1 and IN2. Lineages leading to GR1 and GR2 collapse with LE1 and with lineage leading to VO1 and VO2. Click here for file

Additional file 4 **Topologies derived from Dat-CYB analyses**. A) ML topology, with heuristic search, HKY + G model; 100 random addition sequence. Numbers correspond to bootstrapping frequencies ≥ 60 estimated with 1,000 replicates. Note grouping of *A. lemurinus*/*A. griseimembra *with *A. nancymaae*. B) 50% majority rule consensus topology of 18,000 sampled trees. Numbers at nodes indicate Bayesian proportions. Lineage leading to LE1, GR1 and GR2 collapses with lineage leading to NA1, NA2 and NA3 and with lineage leading to VO1 and VO2. Click here for file

Additional file 5 **Topologies resulting from analyses of deduced aminoacid sequences of *SRY*, *MT-CO1, MT-CO2 *and *MT-CYB***. ML topologies, estimated with heuristic search. Numbers above nodes correspond to bootstrap ≥ 60 estimates with 1,000 replicates. Numbers below nodes indicate Bayesian proportions of 18,000 sampled trees. A) *SRY *with JTT model; B) *MT-CO1 *with mt-mam model; C) *MT-CO2 *with MtMam model, D) *MT-CYB *with MtMam model. Click here for file
