# Supplementary material for: Identification, classification and evolution of Owl Monkeys (Aotus, Illiger 1811)
Source: BMC Evol Biol. 2010 Aug 12;10:248. doi: 10.1186/1471-2148-10-248 (PMC2931504; doi:10.1186/1471-2148-10-248)
Supplement: Additional file 2 — Topologies derived from Dat-CO1 analyses. A) ML topology, with heuristic search, HKY + G model; 100 random addition sequence. Numbers correspond to bootstrapping frequencies ≥ 60 estimated with 1,000 replicates. The lineage leading to NA2 collapses while NA1 and NA3 are grouped. B) 50% majority rule consensus topology of 18,000 sampled trees. Numbers at nodes indicate Bayesian proportions. NA1, NA2 and NA3 are grouped. [file 1471-2148-10-248-S2.PPT]

## Slide 1
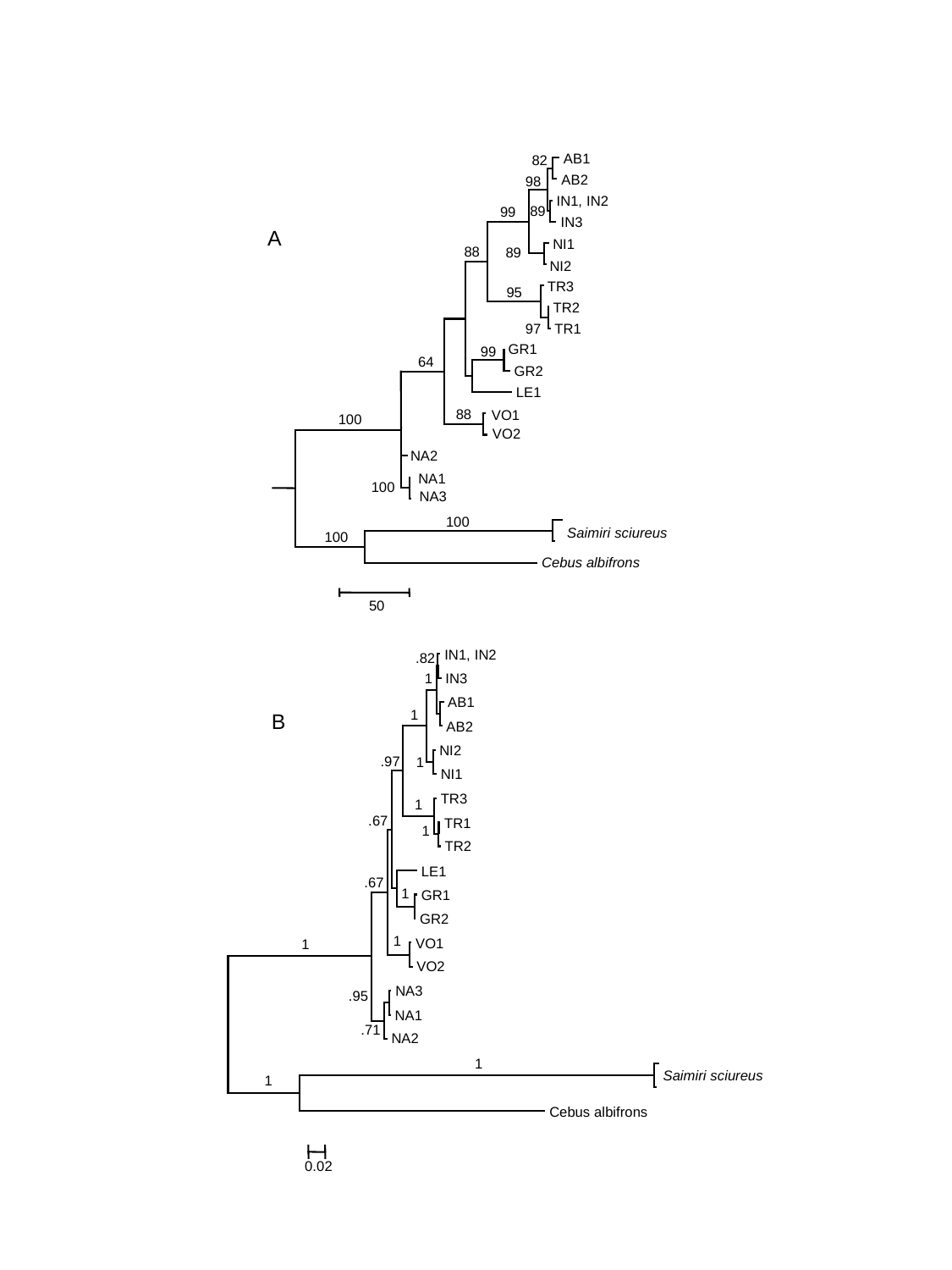

50
AB1
82
AB2
98
IN1, IN2
89
99
IN3
A
NI1
88
89
NI2
TR3
95
TR2
97
TR1
GR1
99
64
GR2
LE1
88
VO1
100
VO2
NA2
NA1
100
NA3
100
Saimiri sciureus
100
Cebus albifrons
 IN1, IN2
.82
 IN3
1
 AB1
1
 AB2
 NI2
.97
1
 NI1
 TR3
1
.67
 TR1
1
 TR2
 LE1
.67
1
 GR1
 GR2
1
 VO1
1
 VO2
 NA3
.95
 NA1
.71
 NA2
1
 Saimiri sciureus
 Cebus albifrons
0.02
B
1
