# Supplementary material for: Identification, classification and evolution of Owl Monkeys (Aotus, Illiger 1811)
Source: BMC Evol Biol. 2010 Aug 12;10:248. doi: 10.1186/1471-2148-10-248 (PMC2931504; doi:10.1186/1471-2148-10-248)
Supplement: Additional file 3 — Topologies derived from Dat-CO2 analyses. A) ML topology, with heuristic search, HKY + G model; 100 random addition sequence. Numbers correspond to bootstrapping frequencies ≥ 60 estimated with 1,000 replicates. IN3 is paraphyletic respective to IN1 and IN2. B) 50% majority rule consensus topology of 18,000 sampled trees. Numbers at nodes indicate Bayesian proportions. IN3 is paraphyletic respective to IN1 and IN2. Lineages leading to GR1 and GR2 collapse with LE1 and with lineage leading to VO1 and VO2. [file 1471-2148-10-248-S3.PPT]

## Slide 1
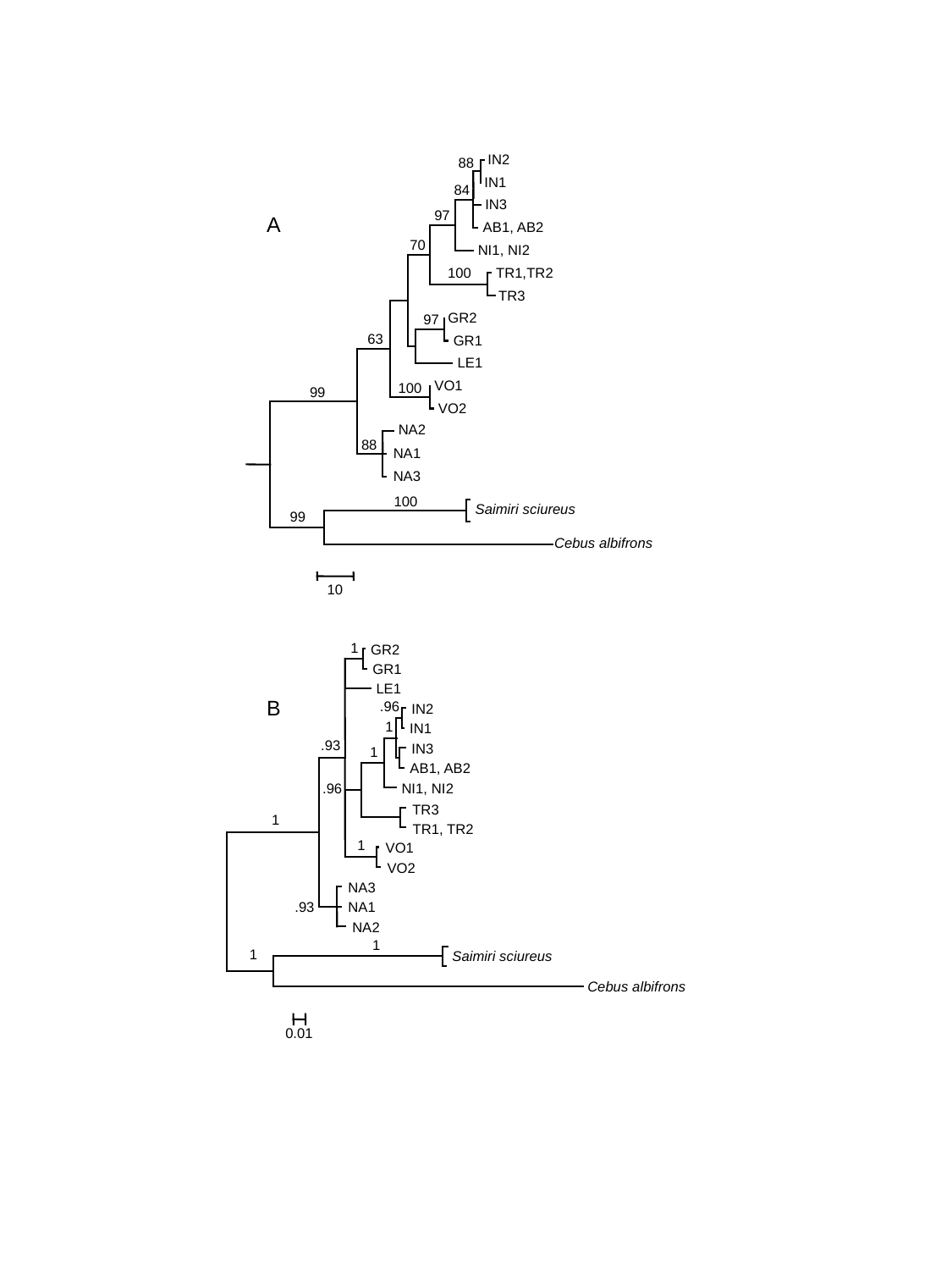

IN2
88
IN1
84
IN3
97
A
AB1, AB2
70
NI1, NI2
100
TR1,TR2
TR3
GR2
97
63
GR1
LE1
VO1
100
99
VO2
NA2
88
NA1
NA3
100
Saimiri sciureus
99
Cebus albifrons
10
1
 GR2
 GR1
 LE1
.96
 IN2
1
 IN1
.93
 IN3
1
 AB1, AB2
.96
 NI1, NI2
 TR3
1
 TR1, TR2
1
 VO1
 VO2
 NA3
.93
 NA1
 NA2
1
 Saimiri sciureus
 Cebus albifrons
0.01
B
1
