# Supplementary material for: Identification, classification and evolution of Owl Monkeys (Aotus, Illiger 1811)
Source: BMC Evol Biol. 2010 Aug 12;10:248. doi: 10.1186/1471-2148-10-248 (PMC2931504; doi:10.1186/1471-2148-10-248)
Supplement: Additional file 4 — Topologies derived from Dat-CYB analyses. A) ML topology, with heuristic search, HKY + G model; 100 random addition sequence. Numbers correspond to bootstrapping frequencies ≥ 60 estimated with 1,000 replicates. Note grouping of A. lemurinus/A. griseimembra with A. nancymaae. B) 50% majority rule consensus topology of 18,000 sampled trees. Numbers at nodes indicate Bayesian proportions. Lineage leading to LE1, GR1 and GR2 collapses with lineage leading to NA1, NA2 and NA3 and with lineage leading to VO1 and VO2. [file 1471-2148-10-248-S4.PPT]

## Slide 1
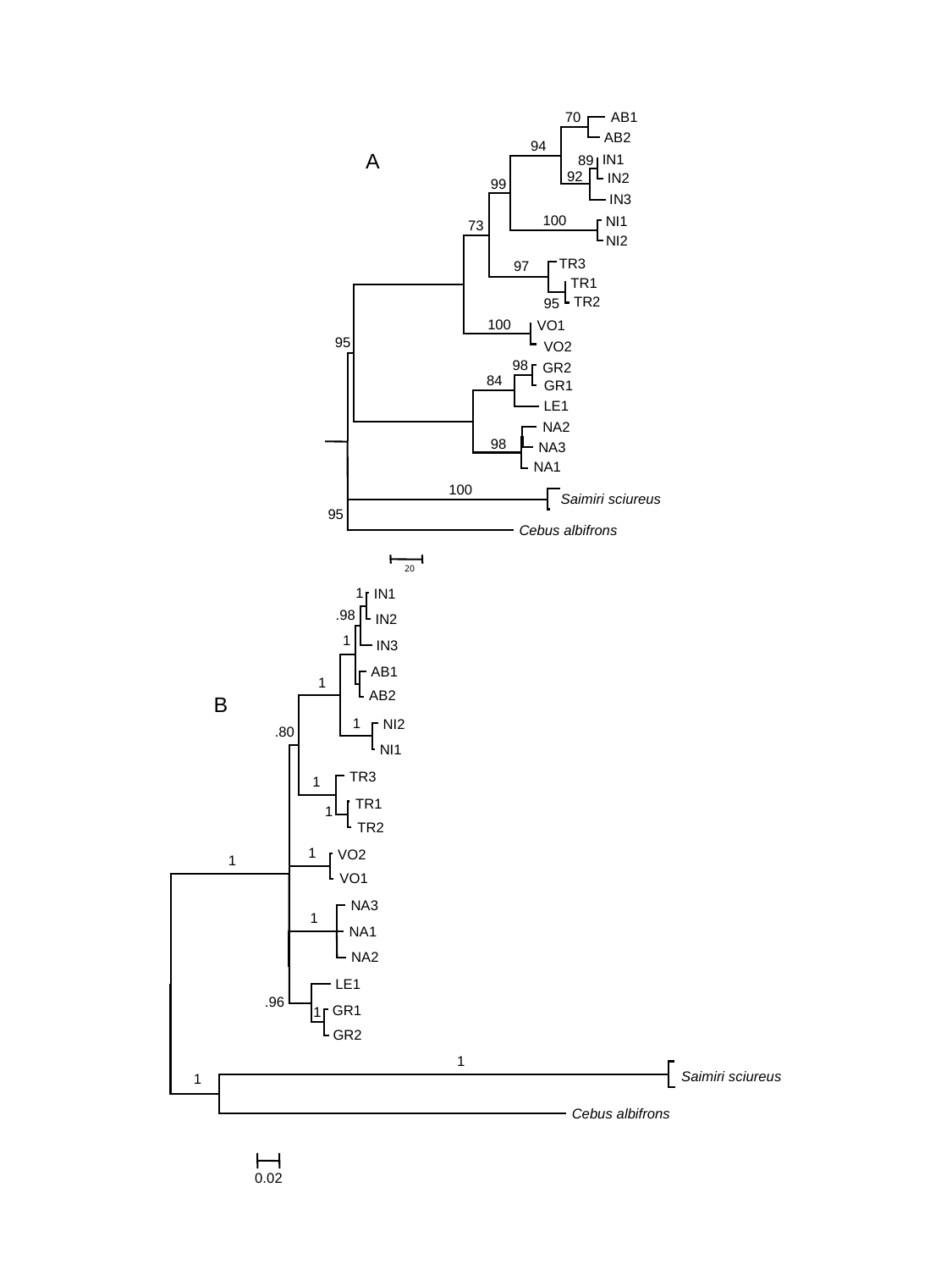

70
AB1
20
AB2
94
A
IN1
89
92
IN2
99
IN3
100
NI1
73
NI2
TR3
97
TR1
TR2
95
100
VO1
95
VO2
98
GR2
84
GR1
LE1
NA2
98
NA3
NA1
100
Saimiri sciureus
95
Cebus albifrons
1
 IN1
.98
 IN2
1
 IN3
 AB1
1
 AB2
1
 NI2
.80
 NI1
 TR3
1
 TR1
1
 TR2
1
 VO2
1
 VO1
 NA3
1
 NA1
 NA2
 LE1
.96
 GR1
1
 GR2
1
 Saimiri sciureus
 Cebus albifrons
0.02
B
1
