# Supplementary material for: Identification, classification and evolution of Owl Monkeys (Aotus, Illiger 1811)
Source: BMC Evol Biol. 2010 Aug 12;10:248. doi: 10.1186/1471-2148-10-248 (PMC2931504; doi:10.1186/1471-2148-10-248)
Supplement: Additional file 5 — Topologies resulting from analyses of deduced aminoacid sequences of SRY, MT-CO1, MT-CO2 and MT-CYB. ML topologies, estimated with heuristic search. Numbers above nodes correspond to bootstrap ≥ 60 estimates with 1,000 replicates. Numbers below nodes indicate Bayesian proportions of 18,000 sampled trees. A) SRY with JTT model; B) MT-CO1 with mt-mam model; C) MT-CO2 with MtMam model, D) MT-CYB with MtMam model. [file 1471-2148-10-248-S5.PPT]

## Slide 1
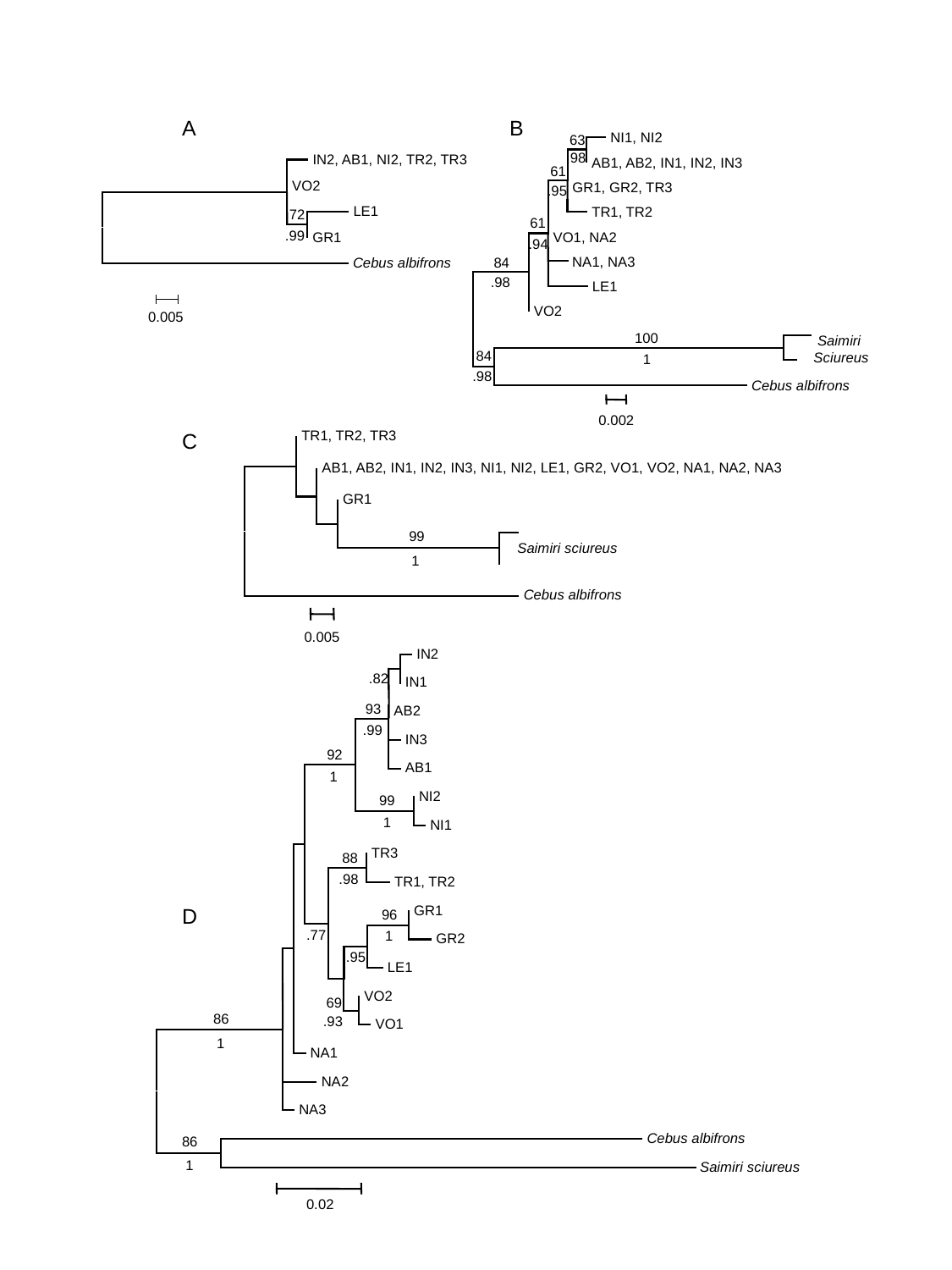

A
 B
 NI1, NI2
63
.98
 IN2, AB1, NI2, TR2, TR3
 AB1, AB2, IN1, IN2, IN3
61
 VO2
 GR1, GR2, TR3
.95
 LE1
 TR1, TR2
72
61
.99
 VO1, NA2
 GR1
.94
 NA1, NA3
84
 Cebus albifrons
.98
 LE1
 VO2
0.005
100
 Saimiri
Sciureus
84
1
.98
 Cebus albifrons
0.002
 TR1, TR2, TR3
 C
 AB1, AB2, IN1, IN2, IN3, NI1, NI2, LE1, GR2, VO1, VO2, NA1, NA2, NA3
 GR1
99
 Saimiri sciureus
1
 Cebus albifrons
0.005
 IN2
 IN1
93
 AB2
 IN3
92
 AB1
 NI2
99
 NI1
 TR3
88
 TR1, TR2
 GR1
96
 GR2
 LE1
 VO2
69
 VO1
 NA1
 NA2
 NA3
 Cebus albifrons
 Saimiri sciureus
0.02
.82
.99
1
1
.98
 D
.77
1
.95
86
.93
1
86
1
